# In Support of Winge's Theory of “Hybridization Followed by Chromosome Doubling”

**DOI:** 10.3389/fpls.2020.00954

**Published:** 2020-06-26

**Authors:** Noemi Tel-Zur, Joseph Mouyal, Udi Zurgil, Yosef Mizrahi

**Affiliations:** ^1^ The French Associates Institute for Agriculture and Biotechnology of Drylands, The Jacob Blaustein Institutes for Desert Research, Ben-Gurion University of the Negev, Beer-Sheva, Israel; ^2^ Department of Life Sciences, Ben-Gurion University of the Negev, Beer-Sheva, Israel

**Keywords:** allopolyploidization, flow cytometry, genome duplication, *Hylocereus*, interspecific-interploid crosses, true hybrids, unreduced gametes

## Abstract

Polyploidy—or chromosome doubling—plays a significant role in plant speciation and evolution. Much of the existing evidence indicates that fusion of unreduced (or 2*n*) gametes is the major pathway responsible for polyploid formation. In the early 1900s, a theory was put forward that the mechanism of “hybridization followed by chromosome doubling” would enable the survival and development of the hybrid zygote by providing each chromosome with a homolog with which to pair. However, to date there is only scant empirical evidence supporting this theory. In our previous study, interspecific-interploid crosses between the tetraploid *Hylocereus megalanthus,* as the female parent, and the diploid *H. undatus,* as the male parent, yielded only allopentaploids, allohexaploids, and 5*x*-and 6*x*-aneuploids instead of the expected allotriploids. No viable hybrids were obtained from the reciprocal cross. Since *H. undatus* underwent normal meiosis with regular pairing in the pollen mother cells and only reduced pollen grains were observed, the allohexaploids obtained supported the concept of “chromosome doubling.” In this work, we report ploidy level, fruit morphology, and pollen viability and diameter in a group of putative hybrids obtained from an embryo rescue procedure following controlled *H. megalanthus* × *H. undatus* crosses, with the aim to elucidate, for the first time, the timing and developmental stage of the chromosome doubling. As in our previous report, no triploids were obtained, but tetraploids, pentaploids, hexaploids, and 5*x*- and 6*x*-aneuploids were found in the regenerated plants. The tetraploids exhibited the morphological features of the maternal parent and could not be considered true hybrids. Based on our previous studies, we can assume that the pentaploids were a result of a fertilization event between one unreduced (2*n*) female gamete from the tetraploid *H. megalanthus* and a normal (*n*) haploid male gamete from *H. undatus*. All the allohexaploids obtained from the embryo rescue technique where those that regenerated from fertilized ovules 10 days after pollination (at the pro-embryo stage), showing that the chromosome doubling event occurred at a very early development stage, i.e., at the zygote stage or shortly after zygote formation. These allohexaploids thus constitute empirical evidence of “hybridization followed by chromosome doubling.”

## Introduction

Genome doubling or polyploidy – the state of having more than two full sets of chromosomes – has played a major role in the diversification and speciation of the plant kingdom, generating the genetic and epigenetic novelty that has contributed significantly to the diversity prevailing today (Stebbins, 1971; Soltis and Soltis, 1993; Osborn et al., 2003; Van de Peer et al., 2017). Autopolyploids arise within a single species and carry homologous chromosomes, while allopolyploids arise from two different taxa and have homoeologous chromosomes (Leitch and Bennett, 1997; Ramsey and Schemske, 1998; Soltis and Soltis, 2000). More specifically, taxonomically, autopolyploids are formed from within a single species, whereas allopolyploids are formed by hybridization between two or more species. Genetically, autopolyploids are plants with random associations among four (in the tetraploid cases) homologous chromosomes, resulting in tetrasomic segregation, whereas allopolyploids have two sets of homoelogous chromosomes that do not typically pair, leading to disomic segregation (Doyle and Egan, 2010; Doyle and Sherman-Broyles, 2017). As such, allopolyploids can, potentially, generate all the enzymes produced by each parent as well as new hybrid enzymes. This genetic redundancy or “enzyme multiplicity” of allopolyploids is considered to be an advantage, contributing to evolutionary success (Soltis and Soltis, 1993; Soltis et al., 2014; Glover et al., 2016; Lloyd et al., 2018) and facilitating speciation when a new hybrid is both fertile and reproductively isolated from its parental species.

The biological significance of allopolyploids thus derives from their origin, establishment, and persistence. Allopolyploid establishment and persistence have been extensively studied over the past few decades (Jenczewski and Alix, 2004; Feldman and Levy, 2009; Steige and Slotte, 2016; and references therein in all these citations), but what do we know about the pathways to the origin and formation of allopolyploids? It was over 100 years ago that Winge (1917) proposed his theory of “hybridization followed by chromosome doubling” as a mechanism enabling the survival and development of the hybrid zygote by providing each chromosome with a homolog with which to pair. According to Winge, hybridization involving two genetically very different gametes would not produce a viable zygote, since the gametes would not be able to pair (as was then solely in the realm of speculation and not based on scientific examinations). He thus suggested that chromosomes would split longitudinally, thereby allowing to generate a pair of homologous chromosomes that would permit the development of the hybrid with double the parental number of chromosomes. However, to date, most empirical studies have not provided evidence to support Winge's theory. Rather, the findings of most studies support one of the following two mechanisms for allopolyploid origin: (1) sexual polyploidization through the fusion of *2n* gametes, where the increase in the chromosome number occurs in the first generation through the union of one or both unreduced gametes (Harlan and deWet, 1975 and references therein, Gao et al., 2019); or (2) chromosome doubling in somatic tissues, i.e., somatic doubling, which is a mitotic rather than a meiotic event. The first report of spontaneous somatic doubling was that for *Primula kewensis*, a first-generation hybrid between *P. floribunda* and *P. verticillata* (Newton and Pellew, 1929). The hybrid diploid plant was sterile, but periodically one of the cuttings produced fertile flowers in branches that were undergoing spontaneous doubling (somatic doubling), a process that apparently restored fertility by providing each chromosome with an identical partner with which to pair (Newton and Darlington, 1929). Today, it is known that somatic doubling in meristem tissues can also be induced by artificial means (Ramsey and Schemske, 1998 and references therein; Sattler et al., 2016 and references therein).

As mentioned above, over the years only scant empirical evidence has been offered in support of Winge's theory of “hybridization followed by chromosome doubling.” To the best of our knowledge, the following examples are the only crosses that support his theory: 1) fertile *Nicotiana glutinosa* L. × *N. tabacum* L. tobacco allotetraploids (Clausen and Goodspeed, 1925); 2) two ryegrass genotypes with an unexpected double number of chromosomes from a BC_2_ progeny [a cross between the diploid *Lolium multiflorum* and a triploid BC_1_ (itself the result of a cross between the tetraploid *Festuca arundinacea* var. glaucescens and a synthetic *L. multiflorum* tetraploid)] (Morgan et al., 2001); and 3) hexaploid and 6*x*-aneuploid pitaya hybrids resulting from a cross between the tetraploid *Hylocereus megalanthus* and the diploid *H. undatus* (Tel-Zur et al., 2003; Tel-Zur et al., 2004). These three examples suggest that the unexpected high ploidy level of the newly formed hybrids was the result of a process that occurred immediately after or soon after fertilization.

*Hylocereus* species are night-blooming vine cacti native to the tropical and sub-tropical regions of the Americas (Barthlott and Hunt, 1993). These species are characterized by triple-ribbed stems, large flowers, and attractive edible fruits (Mizrahi and Nerd, 1999). Cytological studies show that *Hylocereus* species are diploids (Banerji and Sen, 1955; Spencer, 1955; Lichtenzveig et al., 2000; Tel-Zur et al., 2003; Tel-Zur et al., 2004), with the exception of *H. megalanthus*, which is a tetraploid. The diploid species bear large (250-800 g) flavorless, red-purple fruits, whereas the tetraploid *H. megalanthus* bears sweeter, but smaller (180-250 g), yellow fruits (Tel-Zur et al., 2003; Tel-Zur et al., 2011). With the aim of producing elite cultivars with improved fruit quality, a long-term breeding program was thus initiated about three decades ago at Ben-Gurion University of the Negev. In this framework, interspecific-interploid crosses were performed with the aim to combine the size and attractiveness of the diploids with the fruit quality of the tetraploid species (Tel-Zur et al., 2004). No true hybrids or chimeras were obtained in the sampled *H. megalanthus × H. undatus* crosses (Tel-Zur et al., 2003: Tel-Zur et al., 2004), but for the reciprocal cross, tetraploids, pentaploids, hexaploids, and 6*x*-aneuploids, rather than the expected triploids, were obtained (Tel-Zur et al., 2003), thereby indicating a uni-directional gene flow among these species. The tetraploids exhibited the morphological features of the maternal parent and were therefore not considered true hybrids; rather, they were considered to be either a result of self-pollination due to contamination during the hand-cross pollination process or to be of somatic origin, since polyembryony has been reported in *H. megalanthus* (Cisneros et al., 2011). It was therefore proposed that zygotic or post-zygotic somatic chromosome doubling constituted the mechanism for the formation of the hexaploid and 6*x*-aneuploid hybrids (Tel-Zur et al., 2003; Tel-Zur et al., 2004). The above premise was based on our previous work (Lichtenzveig et al., 2000) showing the formation of both unreduced pollen grains and unbalanced gametes due to irregular chromosome disjunction at anaphase. Anaphase I separations such as 22–22, 23–21, and 24–20 were observed in the tetraploid *H. megalanthus*, suggesting that some degree of aneuploidy could be tolerated, while the diploid species exhibited both the regular chromosome disjunction at anaphase I and a uniform pollen diameter (Lichtenzveig et al., 2000; Tel-Zur et al., 2003). Furthermore, to date, all interspecific diploid *Hylocereus* × *Hylocereus* crosses have produced diploid hybrids, which strongly indicates the negligible production of unreduced gametes by the diploid species (Tel-Zur et al., 2004). Consequently, we can assume that the allohexaploid and 6*x*-aneuploid hybrids obtained from crosses of *H. megalanthus* and *H. undatus* occurred at frequencies that were much higher than the frequency that would be expected for the fusion of unreduced gametes from both egg and pollen donor parents. In addition, no chimeras were obtained, i.e., plants with identical fruit and vegetative morphology were observed in the *H. megalanthus* × *H. undatus* hybrids studied, which indicates a very low likelihood of somatic doubling later in development and provides further support for our theory.

Against the above background, the main goals of this research were to confirm the occurrence of spontaneous chromosome doubling following interspecific-interploid hybridization, i.e., Winge's theory, and to identify the timing or the developmental stage at which the event occurs. We postulated that genome doubling takes place either in the hybrid zygote or shortly after zygote formation, a process that ensures the viability of the new hybrid embryo. The methodology applied involved estimating the ploidy level by measuring the relative DNA content by means of flow cytometry of the putative allopolyploids regenerated by the embryo rescue technique following *H. megalanthus × H. undatus* crosses. In addition, the viability and fertility of the resulting true allopolyploids were evaluated in terms of fruit morphology and weight and in terms of the pollen viability and diameter of selected allopolyploids.

## Materials and Methods

### Plant Material and Growth Conditions

The plant material used in this study comprised 38 plants regenerated from crosses between the tetraploid *H. megalanthus* [(Schum. ex Vaupel) Moran] Bauer (accession 90-003) as the female parent and the diploid *H. undatus* (Haw) Br. and Rose (accession 89-024) as the male parent, according to the embryo rescue technique given in Cisneros et al. (2013) and described briefly below. It has previously been shown that the total number of seeds per fruit in *H. megalanthus* is about 200-300, resulting in 20-35% aborted/empty seeds (Cisneros et al., 2011; Tel-Zur et al., 2011). A detailed description of zygote, embryo, and endosperm formation in *H. megalanthus* is provided in Cisneros et al. (2011). In particular, that study reported that an 8-cell pro-embryo was observed in *H. megalanthus* at 11 days after pollination (DAP) and well-developed endosperm was observed 28 DAP. The growing embryo consumed the endosperm, which remained incipient (only a few cells surrounding the embryo) at 42 DAP (Cisneros et al., 2011). Endosperm recovery and/or ploidy determination of endosperm cells is technically very challenging and is thus beyond the scope of our work as discussed below.

The development of the embryo rescue technique in our laboratory started with the rescue of embryos following intraspecific *H. undatus* and *H. megalanthus* crosses. All the resulting offspring showed the expected ploidy, i.e., diploid and tetraploid (unpublished data). In parallel, we applied the embryo rescue technique to fertilized ovules carrying embryos at very early pro-embryonic stage following reciprocal interspecific diploid crosses [*H. undatus* × *H. monacanthus* (syn. *H. polyrhizus*)], which resulted in diploid hybrids alone (Cisneros and Tel-Zur, 2010), thereby showing that the technique did not affect the ploidy level of the resulting hybrids. Briefly, the protocol used for embryo rescue technique included half-strength basal Murashige and Skoog (MS) medium containing 680 µM glutamine, 0.54 µM α-naphthaleneacetic acid, 0.45 µM thidiazuron, and 0.17 M sucrose. The regenerated plants were obtained from fertilized ovules collected from developing fruits at 10, 30, and 47 DAP when the embryos were at the pro-embryo (10 DAP), globular (30 DAP), and heart (47 DAP) stages, respectively, according to Cisneros et al. (2011). The 38 putative allopolyploid plants were planted in January 2013 in 10-L pots held under 50% shade in a greenhouse located on the Bergmann Campus, Ben-Gurion University of the Negev, Beer-Sheva, Israel (31°15'N, 34°48'E). Each plant was watered with 200 L year^-1^ applied *via* a drip system, with a nutrient concentration of 60 mg L^-1^ of N, 18 mg L^-1^ of P and 60 mg L^-1^ of K fertilizer with trace elements (23-7-23 Deshanim, Israel).

### Ploidy Identification

#### Cytological Observations

Developing floral buds were collected from the putative allopolyploid lines and fixed for 24 h in 3:1 ethanol/glacial acetic acid. The buds were stored in 70% ethanol at 4°C until examination. The chromosomes were stained with 2% acetocarmine by using the standard squash method. Pollen mother cells (PMCs) were examined through an Axio ImagerA1 microscope with LED illumination (Zeiss) and photographed with a ZEISS Axiocam 305 color camera and the ZEN imaging software program.

#### Flow Cytometry Analysis

Tissue was collected from the tips of newly developed branches of 4- to 8-year-old putative hybrids, of the allohexaploid J-42, and of the parental species. Nuclear suspensions were prepared from this tissue according to the protocol of Li et al. (2017). Samples were analyzed using a iCyt Synergy SY3200 sorter (Sony Biotechnology, San Jose, USA) equipped with a 561-nm laser and a 595/50 band pass filter. Results were analyzed using Winlist 3D software ver 8.0 (Verity Software House). Genome size was assessed by comparing the nuclear DNA content of the tetraploid *H. megalanthus* with that of the relevant putative allopolyploid. The tissue of each putative hybrid was analyzed at least four times to verify the reproducibility of the results. In addition, a range of nuclear DNA contents indicating each ploidy level was determined on the basis of previously reported nuclear DNA contents for the parental species, i.e., *H. megalanthus* accession 90-003 with 8.70 pg/2C (Tel-Zur et al., 2011), *H. undatus* accession 89-024 with 3.86 pg/2C (Tel-Zur et al., 2011), and the allohexaploid J-42 reported to have 66 chromosomes (Tel-Zur et al., 2004) with 13.4 pg/2C (unpublished data); the ranges of nuclear DNA content per ploidy level were determined according to the variance around the means; thus: 8.00 to 9.50, 10.00 to 11.50, and 12.0 to 13.5 pg/2C for 4*x*, 5*x,* and 6*x*, respectively.

### Fruit Morphology and Weight

Flowers of the putative allopolyploids were cross pollinated by hand at anthesis with a mixture of fresh pollen collected from diploid *Hylocereus* spp. Pollen grains were applied to the surface of the stigmata with the aid of a brush early in the morning. Fruits were collected at the ripening stage of full color over five consecutive years (2014-2018). Fruits from each allopolyploid were weighed on the harvesting day.

### Pollen Grain Stainability and Diameter

Pollen grains from the allopentaploid N-149 and the allohexaploid N-134 were collected at anthesis and stored at 4°C until evaluation. These particular allopolyploids were chosen for this study due to the high number of flowers they produced every year. Pollen grains were stained with 2% acetocarmine (Belling, 1921), since previous studies showed similar outcomes for staining with acetocarmine, fluorescein diacetate, and Alexander's reagent, but acetocarmine can be used to stain both fresh and stored pollen, while the other two reagents are suitable only for fresh pollen. About 300 to 400. and 100 to 150 pollen grains from four different flowers of each line were evaluated for pollen viability and diameter, respectively. Photomicrographs were taken with an Axio ImagerA1 microscope with LED illumination (Zeiss) and photographed with a ZEISS Axiocam 305 color camera and the ZEN imaging software program. Averages ± se were calculated for pollen diameter and pollen viability. A *t*-test was used to assess the differences in pollen performances between allopentaploids and allohexaploids.

## Results

### Ploidy Identification

#### Cytological Observations

**Figure 1:**
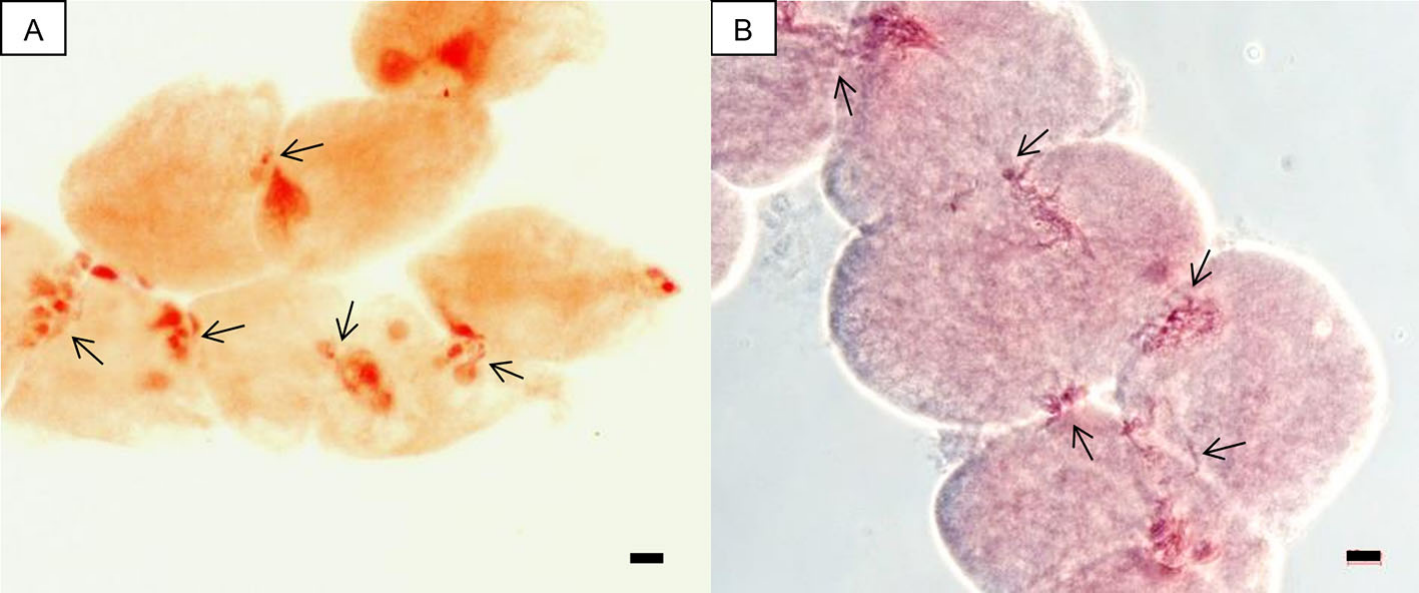
Cytological photographs of groups of PMCs involved in chromatin transfer, i.e., cytomixis (arrow). **(A)** A chain of PMCs united by channels of transferred chromatin in the allopentaploid N-124. **(B)** Migration of chromatin through cytomictic channels in the allohexaploid N-121. Scale bar, 10 µm.

In this study, an attempt was first made to determine ploidy of putative hybrids by chromosome counting. However, cytomixis, i.e., the transfer of chromatin material from one cell to another through intercellular channels (cytomictic channels) during meiosis, proved to be an obstacle that obviated direct counting. Migration of chromatin through cytomictic channels and chains of pollen mother cells (PMCs) united by the migrating chromatin were observed in both allopentaploids and allohexaploids (**Figure 1**). Despite our efforts to very carefully count chromosomes in the PMCs, our results for the number of chromosomes in different PMCs isolated from the same flower bud were not consistent, probably due to the cytomixis. Consequently, ploidy was estimated by flow cytometry.

#### Flow Cytometry Analysis

**Figure 2:**
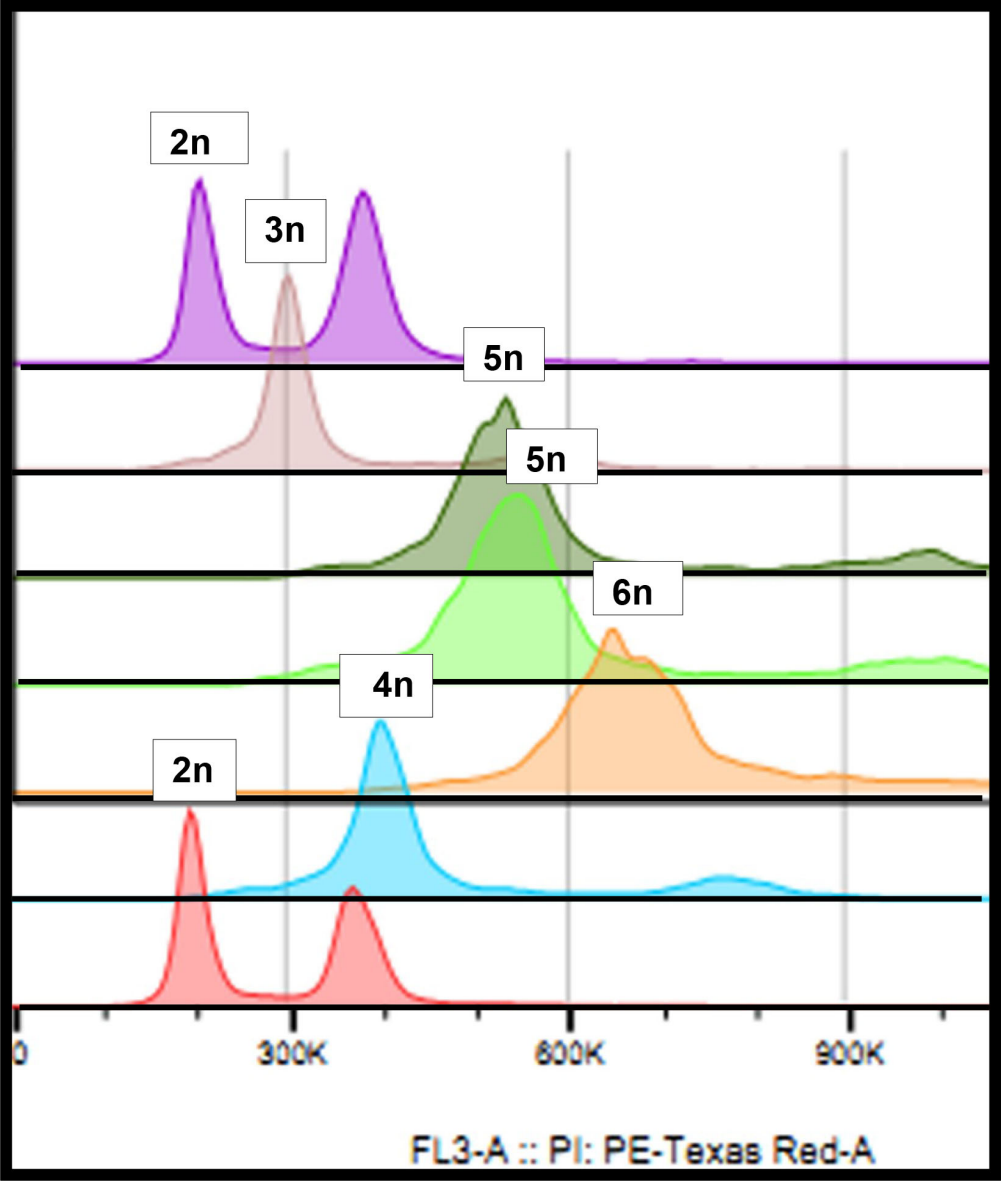
Flow cytometry histogram: violet and dusky pink—the diploid maternal species *H. undatus* at the beginning and the end of the run, respectively; brown—a triploid hybrid (S-75); olive green and light green—two independent runs of the regenerated allopentaploid N-122; orange—the regenerated allohexaploid N-121; and blue—the paternal species, the tetraploid *H. megalanthus*.

The estimated ploidy – calculated as described in Materials and Methods – indicated that among the 38 putative hybrids studied, 32 were true hybrids, and 6 were tetraploids (not considered true hybrids) like the female *H. megalanthus* parent (**Table 1** and **Figure 2**). Of the 32 true hybrids, 18 were allopentaploids and 8 allohexaploids. Nuclear DNA content of the allopentaploids ranged from 10.07 to 11.40 pg/2C, and that of the allohexploids ranged from 12.17 to 13.39 pg/2C (**Table 2**). Estimation of the ploidy level of the other 6 hybrids showed that they exhibited an intermediate ploidy and that they were probably aneuploids, i.e., 3 were 4–5*x*, and 3 were 5–6*x*, ranging from 9.55 to 9.78 pg/2C and 11.67 to 11.76 pg/2C, respectively (**Table 2**). No allotriploids were found in the studied hybrids. Average genome sizes ± se were calculated and are reported for each hybrid line in **Table 2**.

**Table 1:** Ploidy of the confirmed hybrids* according to cytometric analysis.

Cross combination ♀ × ♂	DAP**	No. of progeny planted	No. of confirmed hybrids	No. of confirmed hybrids according to ploidy				
				3*x*	4–5*x*	5*x*	5–6*x*	6*x*
*H. megalanthus* × *H. undatus*	10	19	18	0	1	8	1	8
	30	15	13	0	2	10	1	0
	47	4	1	0	0	0	1	0

*Total number of putative hybrids studied: 38; true hybrids: 32. **DAP, days after pollination.

**Table 2:** Ploidy level estimated using flow cytometric analysis and fruit weight in interspecific-interploid hybrids from a cross of *H. megalanthus* **♀** *× H. undatus* **♂** rescued 10, 30, or 47 days after pollination (DAP) by hand.

Hybrid code	DAP	Nuclear DNA content pg/2C ± se	Ploidy estimated	Fruit weight g ± se
N-121	10	12.98 ± 0.28	6*x*	112.8 ± 16.5
N-122		10.33 ± 0.26	5*x*	124.0 ± 42.8
N-123		13.20 ± 0.32	6*x*	84.2 ± 9.8
N-124		11.30 ± 0.12	5*x*	92.5 ± 12.4
N-132		11.76 ± 0.89	5-6*x*	70.3 ± 12.4
N-133		11.32 ± 0.43	5*x*	100.7 ± 9.2
N-134		12.17 ± 0.28	6*x*	81.8 ± 3.3
N-135		10.72 ± 0.18	5*x*	123.8 ± 6.1
N-136		11.40 ± 0.43	5*x*	133.6 ± 7.0
N-137		11.18 ± 0.54	5*x*	128.8 ± 7.9
N-138		12.69 ± 0.14	6*x*	90.8 ± 6.0
N-140		10.07 ± 0.33	5*x*	110.9 ± 3.6
N-173		10.23 ± 0.35	5*x*	142.7 ± 8.4
N-174		12.65 ± 0.50	6*x*	72.2 ± 4.4
N-175		12.71 ± 0.38	6*x*	62.0 ± 12.3
N-176		13.39 ± 0.31	6*x*	80.9 ± 6.6
N-177		9.55 ± 0.43	4-5*x*	133.0 ± 15.0
N-178		12.54 ± 0.09	6*x*	93.0 ± 3.8
N-125	30	9.78 ± 0.05	4-5*x*	87.6 ± 11.4
N-126		11.29 ± 0.27	5*x*	152.1 ± 10.5
N-127		10.31 ± 0.37	5*x*	154.6 ± 10.8
N-128		11.67 ± 0.32	5-6*x*	123.9 ± 9.5
N-129		10.74 ± 0.43	5*x*	171.0 ± 13.1
N-130		11.18 ± 0.07	5*x*	171.2 ± 10.5
N-131		10.94 ± 0.94	5*x*	147.2 ± 10.7
N-144		11.19 ± 0.32	5*x*	128.1 ± 8.3
N-145		10.52 ± 0.45	5*x*	105.5 ± 7.0
N-147		9.58 ± 0.55	4-5*x*	121.0 ± 6.0
N-148		10.56 ± 0.51	5*x*	139.3 ± 11.7
N-149		11.15 ± 0.45	5*x*	125.9 ± 10.8
N-150		10.89 ± 0.33	5*x*	133.2 ± 10.4
N-013	47	11.74 ± 0.08	5-6*x*	141.8 ± 13.0

### Fruit Morphology and Weight

**Figure 3:**
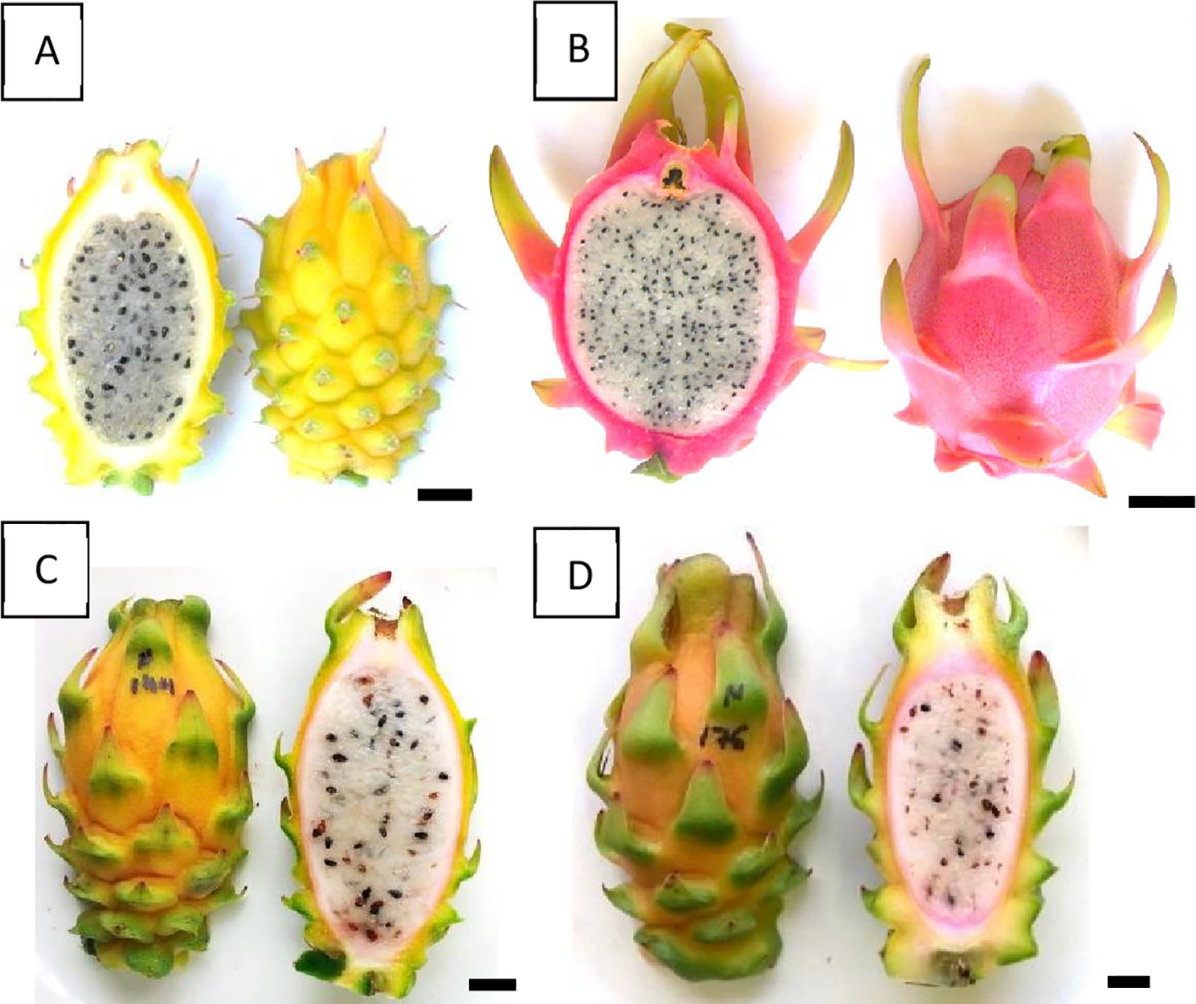
Fruit morphology. **(A)** The maternal species, the tetraploid *H. megalanthus*. **(B)** The paternal species, the diploid *H. undatus*. **(C)** The allopentaploid N-144. **(D)** The allohexaploid N-176. The average fruit weights were 218, 372, 128, and 81 g for *H. megalanthus*, *H. undatus*, allopentaploid N-144 and allohexaploid N-176, respectively.

The putative hybrids developed well under growth conditions similar to those for the parental species. Plant morphology of the putative hybrids, i.e., triple-ribbed stems as well as shape and number of thorns at base of the vegetative buds, was identical to that of the parental lines, indicating that it was impossible to identify true hybrids according to the morphology of the vegetative parts. Fruit morphology of the hybrids was compared with that of the parental species (**Tables 2** and **3**; **Figure 3**). All 32 allopolyploids bore elongated fruits. The fruits were covered by bracts, with thorns at the base of each bract—a trait inherited from the maternal tetraploid species *H. megalanthus*. The spiny peel of all 32 allopolyploids (allopentaploids, allohexaploids, and the probable aneuploids) was yellow-orange in color, with a pink layer between the peel and the flesh. The fruit flesh was white, as in both parental species. All the allopolyploids set both viable and aborted seeds (**Figures 3C, D**).

**Table 3:** Fruit characteristics of the parental species and allopolyploids.

Plant material	Ploidy	Weight, g ± se	Fruit characteristics		
			Spiny peel	Peel color	Flesh color
**Parental species**					
H. megalanthus^*^	4*x*	267 ± 17	+	Yellow	White
*H. undatus* ^*^	2*x*	372 ± 11	−	Red	White
**Cross combination** **♀ × ♂**					
*H. megalanthus × H. undatus*	4-5*x*	ND**	+	Yellow-orange	White
	5*x*	133.5 ± 3	+	Yellow-orange	White
	5-6*x*	ND**	+	Yellow-orange	White
	6*x*	87 ± 3	+	Yellow-orange	White

*Data previously reported by Lichtenzveig et al. (2000) and Tel-Zur et al. (2011). **ND, not determined.

Average fruit weights ± se were calculated and are reported for each hybrid line in **Table 2**. A *t*-test was used to assess the difference in fruit weights between allopentaploids and allohexaploids. Statistically significant differences were observed for fruit weight, as determined by an unpaired two-tailed *t*-test (significant at P = 0.0001), between the allopentaploids and the allohexaploids, with the allohexaploid lines bearing smaller fruit (87 ± 3 g) than the allopentaploid lines (133.5 ± 3 g).

### Pollen Grain Stainability and Diameter

**Figure 4:**
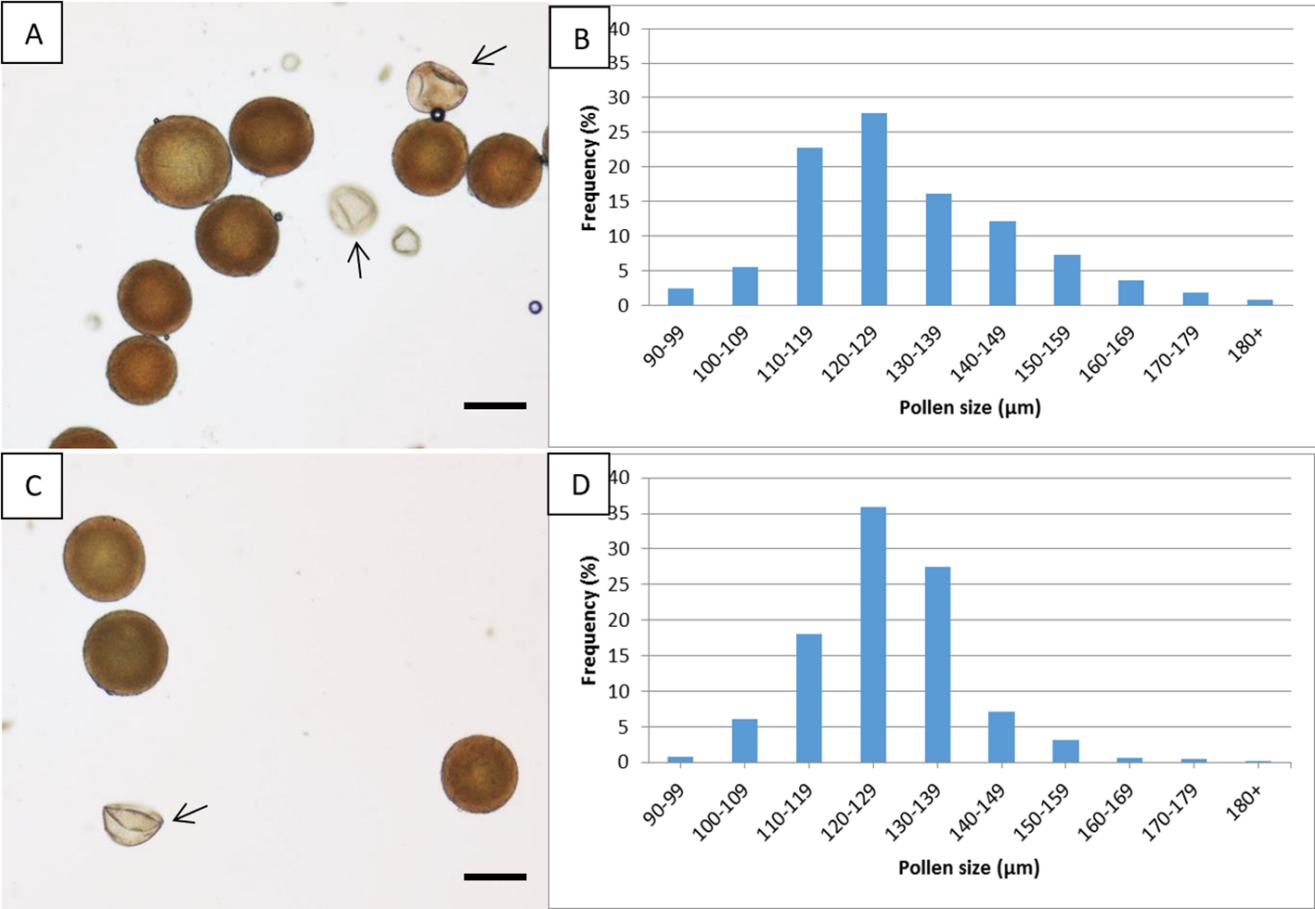
Pollen viability and diameter. Images showing viable and aborted (arrow) pollen grains and pollen diameter distribution in the allopentaploid N-149 **(A, B)** and the allohexaploid N-134 **(C, D)**. Scale bar, 100 µm.

The percent of pollen viability ± se of the allopentaploid N-149 (29.9 ± 0.06%) differed significantly from that of the allohexaploid N-134 (49.5 ± 0.01%), as shown by a *t*-test (significant at P = 0.019). Images showing viable and aborted pollen grains are presented in **Figures 4A, C**. The average ± se pollen diameters were 129.5 ± 0.9 and 126.8 ± 0.6 μm for the allopentaploid N-149 and the allohexaploid N-134, respectively. No statistically significant differences were observed for pollen diameter, as determined by an unpaired two-tailed *t*-test (significant at P = 0.248) between the allopentaploid and allohexaploid lines. The diameters of most of the stainable pollen grains of the allopentaploid N-149 and allohexaploid N-134 (~67% and ~81%, respectively) lay in the range of 110–139 μm, whereas for both allopolyploids, 7% had a smaller diameter than the average, and the remaining diameters exceeded the average range. Pollen diameter frequency distribution is presented in **Figures 4B, D**.

## Discussion

The results of this study strongly support Winge's theory of “hybridization followed by chromosome doubling” by showing high ploidy, i.e., 6*x* instead 3*x*, following interspecific-interploid hybridization. No hybrids were obtained for the *H. undatus × H. megalanthus* cross, and in the reciprocal cross only allopentaploids, allohexaploids, and 4-5*x* and 5-6*x* aneuploids were obtained. It has been proposed that the main route to polyploidization is through unreduced gametes and the formation of triploids (Bretagnolle and Thompson, 1995). The “triploid block” acts as a reproductive barrier in the endosperm, preventing backcrossing with the parental species. Triploids were not obtained for the *H. megalanthus × H. undatus* crosses, but crosses between the diploid *H. monacanthus* as the maternal species and the tetraploid *H. megalanthus* as the paternal species resulted in triploid and 3*x*-aneuploid hybrids, while the reciprocal cross yield only pentaploids (Tel-Zur et al., 2003; Tel-Zur et al., 2004). These findings suggest that *H. monacanthus* is more closely related to *H. megalanthus* than to *H. undatus*. Moreover, back-crosses, using triploid *H. monacanthus × H. megalanthus* hybrids, were indeed obtained (Tel-Zur et al., 2012), thereby showing that the “triploid block” does not exist in *Hylocereus* species.

As mentioned above, we have previously shown that the tetraploid *H. megalanthus* produces both normal (reduced) and unbalanced and unreduced gametes, but the diploid *H. undatus* produces only normally reduced gametes (Lichtenzveig et al., 2000; Tel-Zur et al., 2003). Thus, the presence of allohexaploids and the absence of allotriploids in *H. megalanthus × H. undatus* cross combinations provide empirical evidence supporting our hypothesis that chromosome doubling occurred at the zygote formation stage or very soon thereafter. We thus suggest that chromosome doubling occurs as a spontaneous process of mitotic cell division without cytokinesis. The sources of the 4*x*-5*x* aneuploids are probably unbalanced gametes and unbalanced-unreduced gametes, respectively, from the tetraploid *H. megalanthus* (Lichtenzveig et al., 2000; Tel-Zur et al., 2003). The 6*x* aneuploids are probably a result of a fertilization event with one reduced but unbalanced gamete from *H. megalanthus* followed by chromosome doubling. In this context, it is interesting to note that chromosome doubling at the zygote level can be artificially induced. For example, high temperatures induced the formation of synthetic triploids and tetraploids through embryo sac and zygote embryo chromosome doubling in *Populus simonii* and its hybrids (Guo et al., 2017). Similarly, corn plants exposed to high temperatures after pollination produced diploid, tetraploid, and octoploid seedlings (Randolph, 1932).

One of the crucial questions in allopolyploidy formation relates to the genetic consequences of combining two genetic systems into a common nucleus (Huxley, 1942; McClintock, 1984; Parisod et al., 2009; Lloyd et al., 2018). Subsequent to the successful formation of a new allopolyploid, its establishment depends on the following factors: 1) the correct control of chromosome pairing, a pre-requisite for the production of viable seeds and pollen grains, 2) epigenetic factors, and 3) ‘homoeologous exchanges,' i.e., exchanges of large chromosomal segments between homoeologous chromosomes *via* the meiotic homologous recombination pathway (Jenczewski and Alix, 2004; Feldman and Levy, 2009; Madlung and Wendel, 2013; Glover et al., 2016; Steige and Slotte, 2016; Liang and Schnable, 2018). Natural allopolyploids are stable and well adapted, whereas synthetic allopolyploids can exhibit incompatible interactions between parental genomes, i.e., intergenomic incompatibilities (Comai, 2000). Cytomixis, as was observed here in both allopentaploids and allohexaploids (**Figure 1**), can be the consequence of such intergenomic incompatibilities, but in *Hylocereus* species—like in other species—the biological and evolutionary significance of cytomixis is not known (Mursalimov and Deineko, 2018).

In a parallel but opposite scenario to that reported here, reciprocal crosses between the diploid *Arabidopsis thaliana* and the tetraploid *A. arenosa* resulted in aborted seeds or unsuccessful pollen germination, but crosses between the tetraploid *A. thaliana* as the maternal parent and the tetraploid *A. arenosa* restored seed viability (Bushell et al., 2003). Seeds from reciprocal crosses between the diploid *A. lyrata* and *A. arenosa* failed to germinate, due to failure of endosperm cellularization in the *A. lyrata* × *A. arenosa* cross, while in the reciprocal cross the endosperm cellularized precociously (Lafon-Placette et al., 2017). Interestingly, crosses between the tetraploid *A. lyrate* and the diploid *A. arenosa* were fully viable, but crosses between the tetraploid *A. arenosa* and the diploid *A. lyrate* did not produce viable seeds (Lafon-Placette et al., 2017). Endosperm defects can explain the failure of the cross and the cross direction, illustrating very clearly the role of the endosperm in hybridization barriers.

In flowering plants, sexual reproduction is characterized by two separate gametic fusion events that form the embryo and the endosperm. In diploid crosses, these events result in a diploid embryo and most commonly a triploid endosperm, with a ratio of 1:1 and 2:1 of maternal-to-paternal genomes, respectively. A fundamental question regarding embryo survival and seed development following interspecific-interploidy crosses centers on maternal or paternal genomic excess and the cases in which the embryo and the endosperm can develop with different maternal/paternal genome ratios. To address this question, Johnston et al. (1980) put forward the endosperm balance number (EBN) theory, which stated that rather than an absolute numerical ploidy, each species has an effective genome ratio, which must be 2:1 maternal/paternal for normal endosperm development. This oversimplification of the very important biological phenomenon of zygote formation and embryo development was useful for plant breeders for predicting the success of a cross, especially among *Solanum* species (Carputo et al., 1997; Jansky, 2006), but failed to predict crossing success in other plant species.

We note here that ploidy determination in immature embryos was not directly addressed in this study. *Hylocereus* embryos – even completely mature embryos – are very small, and there is thus not enough tissue to provide sufficient material even for a single flow cytometry run. Nonetheless, even though genome duplication can occur spontaneously in somatic tissues, the probability that such an event would occur in all the studied true hybrids is – in our opinion – extremely low. Moreover, there were no differences between the morphologies of the hybrid plants at the vegetative level and, similarly, no differences in the flow cytometry results for tissues collected from the tips of different newly developed branches; both these findings indicate the low probability of chimera events in the new hybrids.

Determination of endosperm ploidy in the allopolyploids was beyond the scope of this study, but we did calculate the expected endosperm ploidy of the allopentaploid and allohexaploid *H. megalanthus × H. undatus* crosses described above as 9*x* (8m:1p) and 5*x* (4m:1p), respectively, with no production of 3*x* hybrids with an endosperm ploidy of 5*x* (4m:1p) or 10*x* (8m:2p) (**Table 4**). Likewise, no hybrids were obtained in the reciprocal cross *H. undatus* × *H. megalanthus* with an expected endosperm ploidy of 4*x* (2m:2p)—even when the embryo rescue technique was used (**Table 4**). Whole genome duplication (hexaploid rather than triploid) did not change the maternal/paternal ratio in the embryo (**Table 4**), but it did change the maternal-to-endosperm-to-embryo genome ratios by doubling the ploidy of the embryo, which probably allowed normal embryonic development. It therefore seems likely that only the above-described ratios between the endosperm and/or the maternal tissues and/or the embryo permit the survival of the new hybrid embryo in *H. megalanthus × H. undatus* crosses. Studies focusing on the nature of zygotic genome activation have shown that the maternal and paternal genomes contribute equally to early plant embryo development and that early embryogenesis is mostly under zygotic control (Nodine and Bartel, 2012). In rice, for example, polyploid zygotes with a maternal excess developed normally, whereas most polyploid zygotes with a paternal excess showed developmental arrest, indicating that paternal and maternal genomes act synergistically to allow zygote development but probably with distinct functions for each (Toda et al., 2018). The findings that only allopentaploid and allohexaploids were obtained in the *H. megalanthus × H. undatus* cross combination and the lack of allotriploids in both reciprocal crosses suggest that there are (still) unknown factor(s) preventing the formation and development of a triploid embryo.

**Table 4:** Theoretical outcome of interspecific-interploidy crosses following paternal and maternal excess.

	Parental ploidy		Ploidy (m/p)		
	Maternal (m)	Paternal (p)	Predicted endosperm ploidy (m/p)	Hybrid ploidy obtained (m/p)	
**Paternal excess (p)**	2*x*	4*x*	4*x* (2m:2p)		NH**^1^**
**Maternal excess (m)**	4*x*	2*x*	**Normal (reduced) embryo sac**		
			5*x* (4m:1p)		NH**^2^**
			**Unreduced embryo sac** **^3^**		
			9*x* (8m:1p)		5*x* (4m:1p)
			**Normal (reduced) embryo sac followed by zygote/embryo chromosome doubling** **^4^**		
			5*x* (4m:1p)		6*x* (4m:2p)
			**Both endosperm and zygote/embryo chromosome doubling** **^5^**		
			10*x* (8m:2p)		6*x* (4m:2p)

2x: H. undatus, 4x: H. megalanthus.

1 NH - No hybrids were obtained from the H. undatus × H. megalanthus cross, even when we used an embryo rescue technique.

2 NH- No H. megalanthus × H. undatus allotriploids were obtained.

3 Assuming that all the embryo sac cells (including the egg cell) result from unreduced gamete formation, since megasporogenesis occurs before megagametogenesis.

4 Ploidy in the embryo sac cells was assumed to be reduced, while the allohexaploids are a result of chromosome doubling.

5 Chromosome doubling occurs in the endosperm and zygote/embryo, resulting in 10x and 6x, respectively.

*Hylocereus* species bear large flowers (30-35 cm diameter) with numerous mega- and microspores, which simplify technical manipulations (Nerd and Mizrahi, 1997), such as hand cross pollination and the embryo rescue technique (Cisneros et al., 2013). Thus, from a technical point of view, *Hylocereus* species can be used as good model plants in polyploid studies. Among the 32 true hybrids obtained, all eight (25%) allohexaploids were obtained from embryo rescue at 10 DAP (pro-embryo stage), showing that the chromosome doubling event occurs very early in embryo development. We note here that in a previous report (Cisneros et al., 2013) two plants regenerated from embryo rescue 30 DAP following *H. megalanthus × H. undatus* crossing were reported in error as a diploid and a triploid, whereas they were, in fact, tetraploids. Tissues in *Hylocereus* species are rich in polysaccharides, making the flow cytometric analysis a challenging task. Subsequent to that report (Cisneros et al., 2013), and in view of the limitations of the published protocols, we developed an accurate protocol (Li et al., 2017) aimed to solve, simplify, and streamline flow cytometry for ploidy determination in polysaccharide-rich tissues; that procedure was used in this work.

One of the key questions following interspecific and interspecific-interploid hybridization is the fertility of the resulting hybrids. Fruit morphology verifies hybrid origin but also provides the essential information that these hybrids (both allopentaploid and allohexaploid) can grow and can set fruit and seeds. Fruit weights in the allohexaploids were lower than those in the allopentaploids, a finding that is in line with previous reports in auto- and allopolyploid *Hylocereus* species, showing that fruit weight declined as the ploidy level increased (Tel-Zur et al., 2004; Tel-Zur et al., 2012; Cohen et al., 2013). All the allopolyploids set both viable and aborted seeds, and the allopentaploids and allohexaploids produced both viable and aborted pollen grains, showing them to be partially fertile. Pollen viability was about 30% and 50% for the studied allopentaploids and allohexaploids, respectively, with most of the pollen grains presenting – in both cases (67% and 81%, respectively) – a large diameter of 110 to 139 μm. The pollen grains of *H. undatus* exhibited a uniform diameter of 70–80 μm, but those of *H. megalanthus* showed a wide variation in diameter, with values lying between 90 and 190 μm. These differences in diameter represent different ploidy levels in the viable pollen grains and are, most probably, due to meiotic abnormalities, such as the formation of unreduced gametes, unbalanced chromosome segregation, or cytomixis (Ramanna and Jacobsen, 2003; Shamina, 2005; Mursalimov and Deineko, 2018). All the above findings indicate that both allopentaploids and allohexaploids can reproduce by sexual reproduction, since both produce a certain percent of viable male and female gametes. Taken as a whole, our data indicate that interspecific-interploid hybridization among *Hylocereus* does not culminate in a genetic dead-end in the F_1_ generation.

## Conclusions

On the basis of our findings, we concluded that the allohexaploids were produced as a result of “hybridization followed by chromosome doubling” during the very early stage of embryo development, which probably facilitates the survival of hybrids with specific genomic combinations and maternal/paternal ratios in the endosperm.

Interspecific-interploidy *Hylocereus* hybrids constitute a unique model system for the study of polyploidization mechanisms, providing experimental support for somatic chromosome doubling at the zygote or very early embryo development stages, although the nature of the trigger(s) as well as the details of all the process remain unclear. Questions as to the roles of the endosperm in the control of the doubling and in the initial development of the embryo remain open. Further experimental work, including studies on meiotic stability, synapsis, pairing, and recombination, could provide new insights into the possible establishment of these recently produced hybrids with high ploidy.

## Data Availability Statement

The datasets generated for this study are available on request to the corresponding author.

## Author Contributions

NT-Z conceived and planned the experiments. JM and UZ performed the experiments and analyzed the data. NT-Z wrote the manuscript and YM performed part of the experiments and revised the manuscript. All authors contributed to the article and approved the submitted version.

## Conflict of Interest

The authors declare that the research was conducted in the absence of any commercial or financial relationships that could be construed as a potential conflict of interest.
